# Dislocations of Hyoid Bone—Reduction by Kocleus’ Method—Sprains—Their Treatment

**Published:** 1889-06

**Authors:** W. F. Westmoreland

**Affiliations:** Lecturer on Minor Surgery in Atlanta Medical College


VOL. VI. JUNE, 1889. No. 4.

### Original Communications.

### DISLOCATIONS OF HYOID BONE—REDUCTION OF SUB-CORACOID BY KOCLEUS' METHOD—SPRAINS—THEIR TREATMENT.*

BY W. F. WESTMORELAND, JR., LECTURER ON MINOR SURGERY IN ATLANTA MEDICAL COLLEGE.

*Mr. President and Gentlemen of the Association:*

I have been induced to present this irregular paper at this time for the reason that I have lately met with cases illustrating methods of treatment in certain injuries that I have employed with very gratifying results.

The injuries which are discussed in this paper are principally those of articulations. Some of which are important, on account of the frequency with which they are met, and one which is reported for its rarity.

*Read before Medical Association of Georgia, April 18th, 1889.

### DISLOCATION OF THE CORNU OF HYOID BONE—REPORT OF TWO CASES.

Policeman, aged about 28, came into my office complaining of great pain in left side of neck.

Said he had just finished breakfast and while stretching, had thrown his head back, and was yawning, when he suddenly felt a severe pain in left side of neck, and found he could not close his mouth without intense suffering.

His pain was so intense that I had to administer a hypodermic injection of morphine before making an examination.

There was a lump on left side of neck, just over greater cornu. This appeared to be caused as much by the contraction of the muscles as by the displacement of the cornu.

The displacement was upwards and outwards, as near as I could tell.

Laryngoscopic examination revealed no abnormality of either larynx or pharynx. Patient continually complained of sensation that something was sticking in his throat.

The dislocation was easily reduced by having patient throw his head backwards towards opposite side, rubbing, pressing over hyoid bone, and, at the same time, suddenly depressing lower jaw. It was reduced with a *click*, audible to every one in the room, and there was an immediate cessation of all pain. Patient said he had had similar accidents to occur three or four times before; the first time while singing, but has never suffered as much as this time.

He had generally gotten sudden relief while rubbing his throat and moving his head backwards and forwards seeking an easy position. Had always consulted physicians, but they had given him no relief.

CASE II. A young man, traveling salesman from Birmingham, Ala., came into my office seeking relief for his throat. Said he had been in considerable pain for several days, and was unable to keep his mouth closed without increasing this pain; was so inconvenienced by it that he did not think he could attend to his business, as it required a great deal of talking and this increased his suffering. Examination showed lump over right side of hyoid bone, displacement in same direction, but not so great as in former case.

Same manipulations for reduction used as in previous case. Reduced with audible click on first trial with immediate cessation of all pain.

Patient said he had suffered with similar symptoms on several occasions, at long intervals apart, could not remember the exact number.

He had consulted a physician on each occasion; they had invariably prescribed gargles, which he had used.

While using the gargle he would always get relief, sometimes when he first began to use it; again, later, in the following peculiar manner:

While gargling with head thrown back and mouth open, he would be strangled by fluid running down his throat, and, seeking relief, he would bring his head forward to expectorate the medicine.

Just at this time he would feel a peculiar jumping sensation in his throat and hear a slight *click*, and immediately all his unpleasant symptoms would disappear.

In both cases the successive reductions were unconsciously accomplished by the patient going through the manipulation accidentally.

I can only find six cases of this accident recorded in papers on this subject by Dr. Ripley, of South Carolina, and Dr. Gibb, of London, the former reporting one, and the latter five. There is a brief summary of these papers in Holmes' System of Surgery, 2d English edition, vol. II, page 460, and the latter is quoted from in Hamilton's Fractures and Dislocations, 7th edition, page 646.

I do not think that this dislocation is so rare as we would be led to believe by the few cases reported, but that the dislocations are unrecognized, as were the cases I report. And excusably so, for none of the large number of works on surgery that I have examined with reference to this subject, including text books, encyclopædias and special works on fractures and dislocations, with the exception of the two noted above, mention even the possibilities of such an accident.

KOCLEUS' METHOD OF REDUCING SUB-CORACOID DISLOCATIONS OF THE HUMERUS.—REPORT OF TWO CASES.—This injury occurs with much greater frequency than any other dislocation of the body.

Malgaine says that in a total number of 491 dislocations, including all those of the body, 321 were of the humerus.*

Of the 41 specimens of shoulder dislocations preserved in the anatomical museums of London, 31 are sub-coracoid.†

Hulke reports 50 cases of shoulder dislocations seen in practice, 44 of which were sub-coracoid.‡

Chambers' Street Hospital contributes 173 humeral dislocations during the last five years, with 129 sub-coracoid and 44 sub-glenoid; or, out of a total of 274 cases, 203 were sub-coracoid.§

From the above tables the importance of a simple and efficient method of reducing this very frequent dislocation by manipulation can be seen.

CASE I.—A very stout, muscular negro man had his left arm dislocated, by a horse, which he was leading, suddenly rearing and plunging.

Examination showed sub-coracoid dislocation.

Reduced by Kocleus' method in the following manner:

Patient stripped and laid flat on back. I grasped his wrist with one hand, and his arm just above elbow with the other. Flexed his forearm on arm, and adducted his arm to side of body with firm pressure. Holding his arm firmly against side, utilized the lever furnished by the forearm and rotated out; before the rotation was completed, the head slipped easily into the glenoid cavity with crackling sound.

The reduction is not always accomplished this soon; if not, the

*Holmes' System of Surgery, 2d English edition, page 963. Carsten Houlthouse, Art. Inj. of Lower Extremities.

†Same as above, page 870.

‡W. H. Flower and J. W. Hulke, Inj. Upper Extremities.

§Medical Record, March 30, 1889, page 339, Chas. A. Powers, Kochus, etc.

elbow is carried forward and upwards on the chest, with the external rotation, of the arm still maintained and the hand is placed on the sound shoulder. Rarely is more than one trial necessary.

CASE II.—Stout, muscular negro dislocated his shoulder while trying to strike a heavy blow at another man.

Upon examination, diagnosed sub-coracoid dislocation.

Reduced in same manner as in previous case, without any difficulty. The manipulation for description can be divided into four movements.

Place patient in position on back, grasp wrist and arm above elbow.

1st movement: Flex forearm and adduct elbow to body.

2d movement: With elbow still adducted to body, rotate arm until long axis of forearm points directly outwards.

3d: Maintain external rotation of arm and carry elbow forward and upward on the chest.

4th: Place the hand on sound shoulder.

The advantage of this manipulation over Cooper's foot in the axilla, or White's Manchester, 1764, reduction by vertical extension of the arm upwards, is apparent.

### SPRAINS—THEIR TREATMENT.

I have divided sprains into four degrees:

1st. Stretching of the ligament.

2d. Stretching, with partial rupture of the ligament.

3d. Complete rupture of the ligament.

4th. Rupture of a portion of the ligament; the other part, on account of its toughness, is not ruptured, but is torn from its bony attachment, carrying with it the thin shell of bone into which its fibres are inserted. Occasionally none of the fibres are ruptured, the ligament being torn away entire.

This last has been described by Mr. Callender, of London, under the name of sprain-fracture, and by different authors classed as a fracture. But as the sprain is the predominant injury, I have classed it as such, and think this is the nomenclature under which it properly belongs.

Sprains of the first degree, as a rule, need very little treatment beyond the support and elastic pressure that can be given by a well applied flannel roller bandage and perfect rest for a few days.

Occasionally, however, as when a person unconsciously steps down some distance, you may have, besides the twist of ankle, the articular surfaces of the joint driven together with sufficient force to cause a slight contusion of the joint.

When you have a sprain of the first degree, complicated by the contusion, it is best to apply a plaster of paris splint at once; for if the patient is allowed to use the foot, the injury becomes very much aggravated.

Sprains of the last three degrees can, clinically speaking, be classed under the same head; similar treatment being indicated in all.

Lesions which result from these severe sprains differ only in degree from those which attend dislocations. In fact, in sprains of the ankle, you can have such ruptures of the lateral ligaments as in other joints would result in dislocations, but here is prevented by the anatomical construction of the joint, which is intended to entirely prevent any lateral motion. In sprains of the third and fourth degrees there is frequently a slight displacement of the articular surfaces.

The symptoms of these injuries are too familiar to necessitate a description of them here. Indications of treatment are rest and immobility. Unless the surgeon makes it impossible, the patient, finding he can still use the joint, although with pain, will continue to do so until the intenseness of his suffering renders it impossible.

Unfortunately, this is too frequently the case; the patient, thinking he will be confined to bed for several weeks, only seeks the surgeon when he can no longer endure the torture inflicted by using the injured joint.

A well applied plaster of paris bandage makes the best of all splints. It insures perfect rest, immobility and compression of the joint. It should be applied at the earliest possible moment after the injury, whether there is much swelling or not. I recognize no contraindication to its immediate application in these cases. If there is much swelling, the splint can be cut off and a new one applied every few days, as it subsides; or a small piece cut out of the front, and the tightness of the splint regulated by the application of a roller bandage. The success and quickness of the recovery depend on the time that elapses between the receipt of the injury and the application of the splint.

A few words as to the manner of applying the plaster of paris splint. It is not every splint that is properly applied. It is essential that it should be, or it will be uncomfortable, and the patient will not be able to wear it.

The non-success that we hear of in regard to this splint is always due to its being improperly applied by the surgeon, and not to any fault of the splint. If you have bad success with it, abuse yourself, not the splint. First apply a flannel roller bandage; many prefer a close-fitting woolen stocking; the former is better. To draw a stocking over a sprained ankle not only causes pain, but considerable disturbance of the injured parts. Be careful to prevent wrinkling of the bandage, or constriction by its edges being drawn tight, especially in front of the ankle. The former will cause pain, and the latter, swelling of the foot; either will prove sufficient cause for the early removal of the splint.

Place the foot in the proper position (what this is we will discuss later) before the bandage is applied, and turn it over to an assistant, who will hold it steady. Any change in position afterwards is sure to cause wrinkling or tightness of the bandage. Apply the plaster of paris bandage, beginning at a line drawn round the foot at the metatarso-phalangeal articulation, and carrying it up to the bulge at the calf of the leg. Make several extra turns around the joint, carrying them well down around the heel behind, with each bandage; this is where the greatest strain on the bandage will be. While the splint is soft rub it into the concavity on either side of the tendo-achillis, and the irregularities around the two maleoli. It makes the splint hold better. Rub each successive layer thoroughly with your hand as it is applied, to press out the air bubbles, and rub the plaster into the meshes of the crinoline.

There is no rule as to the number of layers to apply. Four layers of bandage, with the extra layers around the ankle, are generally sufficient. I always work in one or two pieces of perforated tin in front of the ankle, to add additional strength at this point, where the greatest strain is.

What is the proper position of the foot? The majority put the foot at right angle to the leg when they apply a splint for this injury. If you will hold your foot at a right angle for a few minutes, you will agree with me that it is awkward, uncomfortable and strained. With a splint on he has to walk flat-footed, which is awkward, tiresome, and necessitates limping. If a shoe is put on the foot, the only part that touches is the heel.

This is a very important question, and on it depends the ability of the patient to walk about comfortably.

When you are in any position that your foot is at rest without touching anything, and both the flexors and extensors are quiet, you will see that the foot is in its natural position of rest. The position is perfectly comfortable, and the foot will remain in it for any length of time without becoming tired; it is the proper one in which to place the foot when the splint is applied. The ball of the foot is on a slightly lower level than the heel.

With the splint on the foot in this position, the patient steps on the ball of the foot, there is some springiness in the walk. As soon as the splint is dry they walk. It is best for them not to use the foot too much for the first few days after the injury.

I have the patient to get what is known as the common sense shoe to wear over the splint. It is a broad toed, laced shoe, with a moderately thick sole and a low, broad heel. With this shoe on, the patient can walk without any perceptible limp and take as much exercise as he wishes.

I have treated four young ladies the past winter, three of them residents of the city, and one a visitor.

CASE I.—A young lady. She hurt her foot nearly a year before I saw her. The injury was evidently a sprain of the second degree, complicated by a contusion of the joint; was unable to walk without crutches. She wore the plaster of paris splints for about six months before she was entirely well, I re-applying them when they cracked or became broken. The patient was able to walk without limping, took all the exercises that were given in a school of physical culture, of which she was a member. She dislocated a toe of the injured foot by kicking a foot ball in the gymnasium. She made a complete recovery in the time mentioned.

CASE II.—A young lady. Saw her twelve days after injury. There was a fracture of the fibula, with complete rupture of the ligament and partial separation of the articular surfaces; ankle terribly swollen; put on splints which she wore for three months; walked without limping; went to germans, dances, etc. and was able to waltz as well, she says, as she could without the splint; would dance the entire programme through.

Case III.—Young lady. Complete rupture of the ligament with partial separation of the articular surfaces. Put on splint within an hour after the injury. Never suffered a particle of pain after the splint was applied. Was able to walk without limping, went to all the dances and waltzed as well as ever. Was able to dance programme through without any inconvenience. Wore splints four weeks.

Case IV.—A young lady in the city, on social visit, accident second day after arrival, when I saw her several hours afterwards. The friends she was visiting were feeling very badly for fear she would be confined to bed and not be able to enjoy the social engagements they had made for her.

There was complete rupture of ligament and partial separation of articular surfaces, greater than in either of the other cases, almost a dislocation. Splint was applied the day after receipt of injury. It was rather late at night when I saw her and I was not able to spare time then on account of serious sickness in my own family, so I put on a couple of flannel bandages.

She went out to a dining in the evening, after having splint applied in the morning, and was able to fill all her social engagements for dinners, drives, theater parties, etc.

She left the city two weeks after splint was applied. I gave her instructions to wear the splint two weeks longer and then take it off.

